# No evidence of a positive effect of learning Chinese language as an L2 on spatial ability

**DOI:** 10.1038/s41598-022-26738-2

**Published:** 2023-01-23

**Authors:** Maxim Likhanov, Olga Bogdanova, Evgenia Alenina, Tatiana Kolienko, Yulia Kovas

**Affiliations:** 1 State Key Laboratory of Cognitive Neuroscience and Learning, Beijing Normal University, Beijing, China; 2 Psychology Department, National Research Tomsk State University, Tomsk, Russia; 3 Laboratory for Social and Cognitive Informatics, National Research University Higher School of Economics, Moscow, Russia; 4 Secondary School Number 43 of Primorski District, Saint-Petersburg, Russia; 5 Department of Psychology, Goldsmiths University of London, New Cross, London, SE14 6NW UK

**Keywords:** Neuroscience, Psychology

## Abstract

Spatial ability (SA) was shown to be a robust predictor of success in various educational contexts, including STEM. Thus, ways to improve SA are of interest to educational psychology. There is some evidence that SA might be improved via learning character-based language, e.g. Chinese as a second language (CSL), however, the existing research is quite limited. The study aims to investigate an effect of CSL learning on SA in schoolchildren from Year 2 to Year 7. Current study employs a sample of Russian schoolchildren (N = 283), who learnt: English only, English and Spanish; or English and Chinese. Participants completed Raven’s progressive matrices and Mental rotation task at the age of 8 and again at the age of 14. Our data showed negligible group differences in the initial SA level at Year 2 (before learning second language). Similar negligible differences were found at Year 7. Regression analysis showed that SA was predicted by intelligence (Raven’s) and gender but not language learnt at both ages. This pattern of results indicates that learning a Chinese as a second language is unlikely to affect SA. Further research is needed to investigate whether other factors, such as length, intensity and context of learning, moderate this link.

## Introduction

Spatial ability (SA) is defined as “the ability to generate, retain, retrieve, and transform well-structured visual images”^1^. It plays an important role in academic performance^2–5^, particularly in interest and accomplishment in Science, Technology, Engineering, and Mathematics (STEM) fields^6–8^. For example, one study^2^ examined the spatial ability data for 563 participants from the Study of Mathematically Precocious Youth (SMPY)^9^. SA, measured at age 13–14, added explanatory power 35 years later: SA accounted for 7.6% of the variance in creative achievement (number of patents and published articles), in addition to the 10.8% of variance explained by scores on the mathematics and verbal sections of the Scholastic Assessment Test. Given that and an ever-growing need of STEM-specialists for modern economy, it is of interest for educational psychology to find ways of SA improvement.

It was shown that SA (and intelligence) grows with age^10,11^. In addition, several studies, including a number of meta-analyses, demonstrated malleability of different SA facets^12^ and transfer of SA training effect to other domains (e.g. maths^13^), using different methods. For example, one study^14^ showed in a randomized controlled design that 6-week-long mental rotation training improves children’s spatial performance, with *η*p^2^ ranging from 0.11 to 0.26. Other methods include videogames with spatial component^12^; sports training^15^; musical training^16^; and origami lessons^17^, with general convention of researchers on the need to “Spatialise the curriculum”^18^.

Several reviews and meta-analyses demonstrated that L2 learning can improve various cognitive abilities, such as working memory, creativity and visuo-spatial ability^19–24^. There is also some evidence that learning of Chinese language as a second language may have positive effect on mathematical learning^10,25^. For example, one longitudinal study^10^ has shown that for Russian sample: (1) spatial ability significantly predicted maths ability (β = 0.180), even after controlling for intelligence; and (2) that there was an effect of language on maths performance (*η*p^2^ = 3%), with participants who learnt Chinese as a second language (CSL) showing higher results than other groups. It is possible that this effect could be attributed to a spatially complex character-based writing system in Chinese language, continuous use of which may continuously train spatial ability (see for discussion^10,26^). Development of spatial ability in turn may have a positive effect on maths ability^3,5,27^.

There is also some evidence from eye-tracking research for improved visuo-spatial perception in people who learn CSL. For example, one study showed a decrease in length of fixations and reduction in number of regressive (backward) saccades in Russian students with increased Chinese language proficiency^28^. Further, several neuroimaging studies have shown that learning Chinese as L2 in adults may lead to increased activation of brain regions involved in visuo-spatial analysis (e.g. bilateral occipital and fusiform areas) for processing orthographical information of Chinese characters^29,30^. This in turn may enhance spatial skill processing more broadly.

Further evidence comes from cross-cultural research that showed higher SA in Chinese native speakers over speakers of other languages^31^. For example, one study^32^ showed that Chinese students demonstrated higher results on five out of 10 spatial tests (cross-sections, pattern assembly, mechanical reasoning, paper folding, and shape rotation) in comparison with Russian students, with Cohen’s d ranging from 0.27 to 0.58. In addition, much research demonstrated advantage of Chinese speakers of all ages in science and mathematics^33–36^. Multiple reasons might be involved in this advantage, including advantage in spatial ability. For example, one study^37^ using a data from PISA for three years have shown that average scores of students from some Asian countries, namely China, Korea and Singapore, are higher on PISA mathematics components compared with other countries. This difference is particularly large for space and shape items of PISA. Moreover, results from PISA demonstrated a robust weak-to-moderate correlation among spatial and other mathematics components. The authors argue that explicit spatial skills instruction could be a means of improving overall mathematical performance. This logic implies that the students from these countries have better spatial skill development opportunities, which may include dedicated educational instruction, but also have a permanent spatial processing inherent in their character based language.

Research has also suggested that SA can be differentially linked to verbal and spatial ability depending on the language^38–41^. For example, effects differed from 0.12 to 0.7 in the aforementioned studies as a function of the language. Such differences may be due to different underlying mechanisms specific to language peculiarities (for example, increased demand for spatial processing). Specifically, for Chinese, the links between verbal and spatial ability were also established, with spatial visualization contributing to word reading over time^42–45^. Moreover, one fMRI study^46^ showed that developmental dyslexia in Chinese is commonly associated with the co-existence of a visuospatial deficit and a phonological disorder.

The current study aims to explore whether learning Chinese versus other second languages affect spatial ability in a sample of Russian schoolchildren, tracked from elementary to secondary school.

The study tests three hypotheses:

1. SA will grow from Grade 2 to Grade 7.
2. Children learning English and Chinese will have higher SA than their peers learning other languages.
3. Chinese learners will show greater improvement in SA from baseline than other languages learners, as reflected in Grade 7 SA score minus Grade 2 SA score.

## Methods

### Participants

283 schoolchildren (Mage = 98.8 months; 55.2 percent females) participated in the current study. In 2012 the first wave of data collection—Wave 1 was completed. Data on spatial ability, intelligence, school achievement (i.e., language learnt in school), demographics (gender, age, etc.) and a number of other measures were collected. Additional 4 data collection waves were completed between 2013 and 2018, using the same instruments. The current study utilizes data collected in Year 2 (Wave 1) and Year 7 (Wave 5), when the children were approximately 8 and 14 years of age. Other variables (e.g. mathematics ability) from an overlapping sample were reported elsewhere^10^.

The data was collected in two specialist language schools, in which schoolchildren are provided with enhanced language curriculum and are able to learn different second languages: (1) English only, (2) English and Spanish; and (3) English and Chinese. In these schools, children begin to study second language earlier with greater frequency of lessons and in smaller groups compared to regular ones. No formal entry criteria were applied in these schools. Schoolchildren and their parents were to choose which language to learn, starting from year two. Year 2 data was collected after approximately 4 months after beginning of second language learning. Year 7 was collected after approximately 5 years of learning second language.

### Procedure

Procedure was slightly different for Year 2 and Year 7

For Year 2, participants completed the tasks as a class exercise during their mathematics lessons under test conditions. Up to two classes were tested per day, so data collection took place over the course of two weeks. After standardized instructions were read to the class, participants were presented with a range of tasks and self-report questionnaires via an online data collection platform (www.dweipsy.com/lattice). The testing took approximately 40 min (see further information in Rodic et al.^25^).

For Year 7, the data were collected online during scheduled classes at school. Only the data for cognitive measures are presented in the current study (see information on other measures in White et al., in preparation). The testing this year also lasted 40 min and was limited by a regular duration of class in Russia.

The research was conducted in schools and the study procedure was approved by each school. The schools appointed a study coordinator, who assisted with all aspects of the data collection. The teacher presented the study to the student’s parents/guardians during the start of the year parents’ meeting. An information sheet on the study was provided and parents/legal guardians had opportunity to ask questions about the planned study. On the day of testing at both Year 2 and Year 7, each child was asked to provide verbal assent if they were willing to participate. All participants were informed that they could withdraw from the study at any time.

### Measures

#### Spatial ability

Mental rotation test^47^ was used to evaluate participants’ spatial ability (specifically, their ability to rotate three dimensional images). In each trial participants are presented with the block of three figures. The target figure is shown on the upper part of the screen and participants have to decide, which of the two remaining figures at the bottom is matching the one at the top by pressing either left or right button. The matching images were rotated from 15° to 345°. Participants have to select the correct answer in as many trials as they could in 3 min. The total number of trials = 181. The task was the same for Y2 and Y7. The final score was calculated by subtracting incorrect responses from correct ones in order to correct for guessing in time-restricted/multiple choice tests, following the procedure described in previous research with this test^25,48–50^. This test has shown good reliability in previous research with Russian sample (Cronbach’s α = 0.87^25^) and in the current one (Split-half reliability = 0.85).

#### Intelligence

Raven’s Progressive matrices^51^ was used to measure general intelligence. It is made of multiple-choice tasks, listed in order of difficulty. Each time participants are presented with an incomplete figure and have to find the missing segment that would complete the figure’s intrinsically regular pattern.

The task differed for Year 2 and Year 7. The total number of items were equal to 80 in Year 2 (parts 12 items from A, and 8 from B, C, D, and E; and 36 items from the Advanced Progressive Matrices). Children had 4 min to complete as many items as they could. Including from both Standard and Advance Raven’s, and restricting time of the test allowed to full spectrum of individual differences at different ages. Total score was computed by subtracting incorrect responses from correct ones to correct for guessing^48–50^. The test showed good reliability in previous studies in UK and Russian samples: Cronbach’s α = 0.67 (N = 154, UK sample) and Cronbach’s α = 0.73 (N = 543, Russia). See Rodic et al., 2018 for a detailed description^49^.

For Year 7 we used a 30-item modification of Raven’s test, excluding all items from sections A and B, including uneven items from sections C, D and E; and including all 12 items from section F. The final score was calculated by summing up all correct responses. This modification of the test was more age-appropriate in terms of difficulty. This test has shown good reliability in previous research with Russian sample (Cronbach’s α = 0.73^25^) and in the current one (Split-half reliability = 0.73).

#### Academic achievement

We used self-reported school Year grades for Russian (native language), English, Spanish and Chinese Language and mathematics (Year grade***). These grades are awarded by teachers to assess a student’s performance for the whole school year in a respective subject (based on performance across the year). The grading system is 1 to 5, where 1 = “terrible/fail”; 2 = “bad/fail”; 3 = “satisfactory”; 4 = “good”; and 5 = “excellent”. A “1” is practically never given, and a “2” is given only rarely, thus the variance in this grade is quite limited. In addition, several recent studies have pointed out that Year grade has a number of limitations, e.g. teacher bias towards students’ personality; and inability to distinguish between populations selected for high-achievement in Science and their peers (see Likhanov et al.^54^, Papageorgiou et al.^55^ and Budakova et al.^40^, for a discussion of the limitations of this grading system). See Appendix for descriptive statistics and correlations for these Year grades.

All analysis was performed in the R statistical software package^54^ and JASP statistical package^55^.

### Ethical approval

The study was approved by the Psychology Department Ethical committee, Goldsmiths, University of London (code of ethical approval: PS020813YKST) and TSU Ethics Committee for Interdisciplinary Research (code of approval: 01162017-4) and was carried out in accordance with their recommendations and the Declaration of Helsinki. An informed consent was obtained from parent/legal guardians of participants.

## Results

### Descriptive statistics and correlations

Descriptive statistics for study variables are presented in Table 1 for the overall sample and in Table 3, divided by language schoolchildren learn. The means for spatial ability were different between Year 2 and Year 7, with t (120) = 4.58, *p* < 0.05 (Cohen’s d = 0.42; see Table 1 for means and SDs). Correlations for the study variables are presented in Table 2.

**Table 1:** Descriptive statistics for all study variables.

	SA Year 2	SA Year 7	SA growth*	Raven’s Year 2^+^	Raven’s Year 7^+^
N	188	186	121	180	198
Mean	12.16	16.49	7.19	5.86	9.92
Std. Deviation	9.20	15.96	17.27	3.28	4.12
Minimum	− 14.00	− 22.00	− 35.00	0.00	0.00
Maximum	34.00	56.00	54.00	13.00	22.00

SA Growth was computed by subtracting SA Year 2 from SA Year 7; Paired sample t-test was applied to the data, comparing Year 2 versus Year 7 cognitive measures. Effects were significant for both SA and Raven’s; ^+^ as the versions of the Ravens’ test differed between Year 2 and Year 7, the means are not directly comparable.

**Table 2:** Correlations for main study variables.

Variable		SA Year 2	SA Year 7	Raven’s Year 2	Raven’s Year 7
1. SA Year 7	n	121	–		
	Pearson's r	0.153	–		
2. Raven’s Year 2	n	176	117	–	
	Pearson's r	0.334***	0.254**	–	
3. Raven’s Year 7	n	129	186	125	–
	Pearson's r	0.054	0.421***	0.044	–
4. SA growth	n	121	121	114	121
	Pearson's r	− 0.439***	0.821***	0.075	0.199*

*p < 0.05, **p < 0.01, ***p < 0.001; SA Growth was computed by subtracting SA Year 2 from SA Year 7.

### Predicting spatial ability from language group

In order to check the effect of language on spatial ability, 5 regression models were computed: 2 predicting spatial ability in Year 2 and 3 predicting spatial ability in Year 7. Given the consistent gender differences reported for spatial ability (see, for example studies^3,32,56^), gender was added to all models. Two dummy code variables for L2 language were introduced for this analysis and were coded as follows: language code 1: English only = 0 (reference group, as all students learned English), English and Spanish = 1, English and Chinese = 0; language code 2: English only = 0 (reference group), English and Spanish = 0, English and Chinese = 1. These two variables were then used as predictors in regression models. Results are presented in Table 4.

**Table 3:** Descriptive statistics for each study variable divided by language and group comparisons.

Language	SA Year 2			SA Year 7			SA Growth			Raven’s Year 2^+^			Raven’s Year 7^+^		
	1	2	3	1	2	3	1	2	3	1	2	3	1	2	3
N	66	61	61	87	67	32	51	48	22	62	59	59	95	68	35
Mean	10.24	11.61	14.80	13.22	18.85	20.48	5.61	8.87	7.18	4.32	5.27	8.07	9.05	10.85	10.49
Std. Deviation	9.57	9.84	7.48	16.55	15.41	14.04	19.25	16.54	14.03	2.57	2.68	3.34	3.88	4.08	4.44
Minimum	− 10.00	− 14.00	− 2.00	− 19.00	− 22.00	− 5.00	− 35.00	− 35.00	− 26.00	1.00	1.00	0.00	0.00	0.00	0.00
Maximum	34.00	30.00	33.00	56.00	47.00	49.00	54.00	43.00	30.00	12.00	12.00	13.00	20.00	22.00	20.00
ANOVA (Patial eta squared)	0.04*			0.04*			0.01			0.24***			0.04*		

*p < 0.05, **p < 0.01, ***p <0 .001; Language variable is coded as follows: 1—English only; 2—English and Spanish; and 3—English and Chinese; SA Growth was computed by subtracting SA Year 2 from SA Year 7; ^+^ as the versions of the Ravens’ test differed between Year 2 and Year 7, the means are not directly comparable. For Year 2 total scores were computed by subtracting incorrect responses from correct responses—guessing correction (see Methods section). For Year 7 a slightly different Raven’s test (adjusted for age) was used, with total scores computed by summing up all correct items.

**Table 4:** Linear regression predicting spatial ability from gender, language and intelligence in Year 2 and Year 7.

IV + :	DV: SA Year 2		DV: SA Year 7			DV: SA Growth
	Model 1	Model 2	Model 3	Model 4	Model 5	Model 6
Gender++	3.15*	2.78*	7.65***	8.94**	8.78**	6.08
Language code 1	1.94	1.55	6.48*	3.71	3.28	2.64
Language code 2	5.39**	2.18	7.68*	1.94	1.47	-0.21
Raven’s Year 2	–	0.85*** [0.31***]	–	1.51** [0.25**]	1.38* [0.22*]	0.41 [0.65]
Raven’s Year 7	–	–	–	–	1.11** [0.26**]	0.95* [0.20*]
R^2	0.07	0.14	0.09	0.14	0.17	0.04
F (df)	4.68 (3,184)**	6.84 (4,171)***	6.39 (3,182)***	5.97 (3,113)***	5.83 (5,111)***	1.89 (5,109)
N	188	176	186	117	117	114

*p < 0.05, **p < 0.01, ***p < 0.001; + unstandardized coefficients are presented for IVs’ in each model, except Raven’s test for which standardized coefficients are presented in square brackets []; ++ gender was coded as follows: 0—female, 1—male, thus, positive effect should be interpreted as males demonstrating higher SA than females; SA Growth was computed by subtracting SA Year 2 from SA Year 7.

Our data showed that for Year 2 spatial ability was predicted by gender and language code 2 with moderate effects (Model 1), but effect of language disappeared when Raven’s score for Year 2 was added to the model (Model 2). The same pattern was shown for spatial ability measured in Year 7. Gender and language group (code 1 and code 2) predicted SA in Year 7 (Model 3); but the effect of language disappeared when Raven’s measured in Year 2 was added (Model 4). The same pattern persisted when Raven’s measured in Year 7 was also added to the model (Model 5).

### Spatial ability growth

We conducted an additional analysis to explore predictors of growth in spatial ability, computed by score subtracting SA Year 2 from SA Year 7 (see Table 1 and Table 3 for descriptive statistics and Table 2 for correlations with other variables). Our data showed that SA growth was linked only to intelligence at Year 7 (see Model 6 in Table 4).

## Discussion

The aim of the current study was to explore spatial ability in schoolchildren, tracked from elementary to secondary school.

Our data supported the first hypothesis: spatial ability increased with age—from Year 2 to Year 7. These results add to the existing literature that suggests a steady increase in spatial ability across development^10,57^. However, the exact mechanisms of this growth are very difficult to identify. Multiple predictors of SA growth were suggested in the literature, including number sense^58^, interests in spatial activities^59^, and self-perception^60^.

Contrary to our prediction no positive effect on spatial ability was found for learning Chinese as a second language. Our data showed that while spatial ability did vary as a function of language in both Year 2 and Year 7 (β equal to 0.24 and 0.20, respectively); this link disappeared after intelligence in Year 2 was added to the model. Thus, we did not find a positive transfer effect of Chinese language learning on spatial ability. One potential explanation for absence of the effect after controlling for intelligence is that parental decisions regarding the choice of school and second language may be influenced by their perceptions of their children’s cognitive ability. Despite the absence of school entry selection, parents whose children experience difficulties with preschool educational programme might have opted for easier option—learning of only one second language, namely English. At the same time children with greater preschool learning indicators (e.g., early number knowledge) could have decided to learn two second languages (either English and Spanish, or English and Chinese) and thus, demonstrated on average higher intelligence both in Year 2 and Year 7.

Our results on differences between language group in intelligence are consistent with differences in achievement, intelligence and other psychological characteristic found between selected and unselected for prior achievement groups^40,61,53^. For example, one study^52^ showed that STEM-selected adolescents demonstrated higher year grades and state exams results (*η*p^2^ = 0.05 and 0.23, respectively), compared with their unselected peers.

This explanation is in line with a very well-established link between different measures of academic achievement and intelligence in schoolchildren (0.37–0.94^62–64^). Such links has also been demonstrated between spatial ability and academic achievement, with stronger links with maths than verbal ability^3,40,61,65^.

Parental-choice explanation might be especially true for choosing between learning only English and English plus Chinese, as: (1) English only group was significantly lower on Raven’s compared with English + Chinese group; with no differences between English only and English + Spanish group at Year 2; and (2) Chinese language was previously shown to be perceived as more difficult to learn, compared with Western languages, i.e., English or Spanish^66–70^.

Our third hypothesis was also not supported: Chinese learners did not show greater improvement in SA from baseline (Year 2) to Year 7 compared with learners of other languages. Regression analysis showed that spatial ability grows irrespective of language learned, sex and intelligence in Year 2. The increase in spatial ability may be explained by similar mechanisms that underlie grows in “g”. For example, one meta-analysis showed intelligence growing from 1 to 5 IQ points for an additional year of education^71^. Spatial ability is considered to be a part of general cognitive ability (“g”^72^), which is consistent with the pattern of correlations in the current study (0.26–0.46).

Differences in Raven’s scores across the language groups were higher at Year 2 compared with Year 7 (0.24 vs. 0.04). Smaller effect at Year 7 might be linked to school education acting as an equalizer, reducing the gaps in intelligence, existing at the beginning of the formal education. This pattern of results is in line with a wealth of previous research^73–75^. Alternatively, this pattern of results might be driven by the changes in the way Raven’s scores were computed. For Year 2 total scores were computed by subtracting incorrect responses from correct responses—guessing correction (see Methods section). For Year 7 a slightly different Raven’s test (adjusted for age) was used, with total scores computed by summing up all correct items. In addition, this pattern of results might be due to changes in the number of study participants between Year 2 and Year 7. Specifically, number of participants who completed spatial ability test: increased for English (from 66 to 87) and English + Spanish group (61–67); and reduced for Chinese group (61–32). These changes might be attributed to sample attrition (changing of school or missing school on the date of testing); and limited time of testing (not all children were able to complete all tests).

Apart from testing the main hypotheses, we were able to investigate sex differences in SA. The results of the study found further support for male advantage in spatial ability—an effect robustly demonstrated in the literature^40,56,76,77^. In contrast, no sex differences were observed for Raven’s progressive matrices– again consistent with recent research^78^. We also conducted a supplementary sex differences analysis to further explore links between Chinese language and spatial ability (see Table S1 in Appendix). Specifically, we tested whether males would demonstrate greater achievement in Chinese language compared to other languages. This would potentially demonstrate a positive effect of higher spatial ability on acquisition of Chinese spatially-complex writing system. Our data showed that girls outperformed boys in all languages, replicating a well-established female language advantage^79–81^. Contrary to our expectations, girls’ advantage was even greater in Chinese language compared to other languages, suggesting absence of positive effect of higher spatial ability on Chinese language acquisition. More research with bigger samples is needed to investigate both directions of effect: contribution of spatial ability to character-based languages learning and contribution of learning character-based languages to spatial ability development.

To further investigate the links between spatial ability and Chinese language proficiency, we conducted an additional analysis, using the only available achievement measure—a Year grade. By the end of Year 7 the mean Year Grade in Chinese was 4.01, which indicates a good proficiency according to the school programme (the highest Year Grade is 5). In addition, we found that overall achievement in Chinese language has decreased slightly from Year 2 to Year 7 according to this measure, which is consistent with previous research investigating transition from primary to secondary school^82^. There were no correlations between: (1) Year grade in Chinese and spatial ability for Year 2 and Year 7; and (2) the growth in spatial ability was not correlated with changes in Year grade (see Tables S2 and S3 in Appendix). The absence of correlation may be a result of a small sample in which the analysis was conducted (n varied from 27 to 61). In addition, this pattern of results might reflect crudeness of the Year grade: (1) the measure has only 4 possible levels (2–5), with 2 (fail) being extremely rare; and (2) the grade is not standardized across different schools/classes (for example, a 3 awarded to mathematically gifted student, attending a specialized mathematics class, is not equal to their peers from a different school; see Likhanov et al.^54^ for a discussion). Crudeness of this measure precluded us from investigating specificity of the L2 learning effect on spatial ability for example by adding additional covariates (e.g. maths knowledge) and outcomes, such as school achievement in different subjects, to regression models. In addition, in our study the Year grades are strongly correlated with each other, which also precluded a fine-grained investigation (See Table S3). For example, Year grade in mathematics correlated 0.72 with Russian language on Year 2, which is in line with much previous research (see e.g.^83^).

## Conclusions and future directions

Overall, the results of the study suggest no positive effect of learning Chinese as a second language on spatial ability. More research is needed as current study has a number of limitations. Firstly, there was an attrition between Year 2 and Year 7 in English and Chinese group. There may be multiple reasons for this attrition, including children not being present on the day of testing; children switching schools (possibly to a school with one second language); etc. In order to properly investigate whether attrition was random a larger sample is needed. Secondly, the current study assessed language x spatial ability link over a span of 5 years, demonstrating zero effect of language. However, the effect of language might be subtler and emerge only on a bigger time scale (10–20–30 years) or with more intensive engagement with language (e.g., living in China) or only at highest language of proficiency. It is also possible that the effect is only present in native speakers, who are continuously engaged with Chinese language from an early age. Thirdly, in the current study we used two different versions of the Raven’s which could not be directly compared. Nevertheless, the inclusion of Raven’s in each age was useful to get insights into potential school selection processes. Fourthly, our study investigated an effect of Chinese language only on spatial ability, whereas an effect may emerge for other cognitive abilities, such as visuo-spatial working memory, auditory perception and long-term memory.

Finally, a more comprehensive investigation of the reciprocal links between Chinese language and spatial ability is needed, including tracking longitudinally contribution of spatial ability to Chinese acquisition and vice versa. Further research in this area will help to bridge the current understanding of key role of spatial ability in cognitive processing and harnessing the power of spatial ability for improving educational outcomes.

## Supplementary Information

Supplementary Information.

## Data Availability

The data that support the findings of this study are available from the corresponding author upon reasonable request.
