# Supplementary material for: No evidence of a positive effect of learning Chinese language as an L2 on spatial ability
Source: Sci Rep. 2023 Jan 23;13:1262. doi: 10.1038/s41598-022-26738-2 (PMC9871025; doi:10.1038/s41598-022-26738-2)
Supplement: Supplementary file 1 — Supplementary Information. [file 41598_2022_26738_MOESM1_ESM.docx]

**Title: No evidence of a positive effect of learning Chinese language as an L2 on spatial ability**

**Authors:** Maxim Likhanov ^1^, Olga Bogdanova^2^, Evgenia Alenina^3^, Tatiana Kolienko^4^, and Yulia Kovas^5*^

^1^ State Key Laboratory of Cognitive Neuroscience and Learning, Beijing Normal University

^2^ Psychology Department, National Research Tomsk State University

^3^ Laboratory for Social and Cognitive Informatics, National Research University Higher School of Economics

^4^ Secondary school number 43 of Primorski district, Saint-Petersburg

^5^ Department of Psychology, Goldsmiths University of London, UK

*Corresponding author information: Yulia Kovas, Department of Psychology, Goldsmiths University of London, New Cross, London, SE14 6NW, UK; email: y.kovas@gold.ac.uk.

**Appendix**

| **Table S1 Descriptive Statistics for all study variables divided by sex** | | | | | | | | | | | | | | | | | | | | | | | | | | | | | | | | | | | | | | | | | | | |
| --- | --- | --- | --- | --- | --- | --- | --- | --- | --- | --- | --- | --- | --- | --- | --- | --- | --- | --- | --- | --- | --- | --- | --- | --- | --- | --- | --- | --- | --- | --- | --- | --- | --- | --- | --- | --- | --- | --- | --- | --- | --- | --- | --- |
|  | | **Spatial ability** | | | | | | | | | | | **Raven’s test** | | | | | | | | | | | | **School marks at Year 7** | | | | | | | | | | | | | | | | | | |
|  | | **Year 2** | | | | **Year 7** | | | **Growth** | | | | **Year 2** | | | | | | **Year 7** | | | | | | **English** | | | | | | **Chinese*** | | | | | | **Spanish**** | | | | | |  |
|  | | **f** | | **m** | | **f** | **m** | | **f** | | **m** | | **f** | | | **m** | | | **f** | | | **m** | | | **f** | | | **m** | | | **f** | | | **m** | | | **f** | | | **m** | | |  |
| N |  | 107 |  | 81 |  | 97 |  | 89 | 64 | 57 | |  | | 101 |  | | 79 |  | | 103 |  | | 95 |  | | 132 |  | | 107 |  | | 42 |  | | 31 |  | | 50 |  | | 33 |  |  |
| Mean |  | 11.21 |  | 13.42 |  | 13.12 |  | 20.16 | 5.27 | 9.35 | |  | | 6.14 |  | | 5.51 |  | | 9.64 |  | | 10.23 |  | | 4.11 |  | | 3.73 |  | | 4.29 |  | | 3.64 |  | | 4.50 |  | | 4.15 |  |  |
| Std. Deviation |  | 7.84 |  | 10.65 |  | 15.86 |  | 15.32 | 17.20 | 17.25 | |  | | 3.41 |  | | 3.08 |  | | 3.96 |  | | 4.28 |  | | 0.76 |  | | 0.69 |  | | 0.64 |  | | 0.66 |  | | 0.65 |  | | 0.75 |  |  |
| Skewness |  | -0.51 |  | -0.24 |  | -0.03 |  | -0.28 | -0.17 | -0.45 | |  | | 0.31 |  | | 0.70 |  | | -0.07 |  | | 0.15 |  | | -0.18 |  | | 0.40 |  | | -0.32 |  | | -0.21 |  | | -0.94 |  | | -0.26 |  |  |
| Kurtosis |  | -0.21 |  | -0.52 |  | -0.93 |  | -0.46 | -0.29 | 0.91 | |  | | -0.97 |  | | -0.06 |  | | -0.09 |  | | 0.18 |  | | -1.26 |  | | -0.86 |  | | -0.60 |  | | 0.14 |  | | -0.14 |  | | -1.15 |  |  |
| Minimum |  | -12.00 |  | -14.00 |  | -22.00 |  | -15.00 | -35.00 | -35.00 | |  | | 1.00 |  | | 0.00 |  | | 0.00 |  | | 0.00 |  | | 3.00 |  | | 3.00 |  | | 3.00 |  | | 2.00 |  | | 3.00 |  | | 3.00 |  |  |
| Maximum |  | 25.00 |  | 34.00 |  | 47.00 |  | 56.00 | 43.00 | 54.00 | |  | | 13.00 |  | | 13.00 |  | | 20.00 |  | | 22.00 |  | | 5.00 |  | | 5.00 |  | | 5.00 |  | | 5.00 |  | | 5.00 |  | | 5.00 |  |  |
| t-test: t |  | -1.63 | | |  | -3.07 | | | -1.30 | | |  | | 1.29 | | | |  | | -1.01 | | | |  | | 3.86 | | | |  | | 4.185 | | | |  | | 2.24 | | | |  |  |
| t-test: df |  | 186 | | |  | 184 | | | 119 | | |  | | 178 | | | |  | | 196 | | | |  | | 237 | | | |  | | 71 | | | |  | | 81 | | | |  |  |
| t-test: Cohen’s d |  | -0.24 | | |  | -0.45 | | | -0.24 | | |  | | 0.19 | | | |  | | -0.14 | | | |  | | 0.50 | | | |  | | 0.99 | | | |  | | 0.50 | | | |  |  |
| p-value |  | ns | | |  | .002 | | | ns | | |  | | ns | | | |  | | ns | | | |  | | <.001 | | | |  | | <.001 | | | |  | | .027 | | | |  |  |
| *Note.*   * only available for English and Spanish group; ** only available for English and Spanish group | | | | | | | | | | | | | | | | | | | | | | | | | | | | | | | | | | | | | | | | | | | |

**Year grades analysis**

Year grade for Chinese language correlated between Year 2 and Year 7, with Pearson’s r equal to .46. The means for Chinese language year grade were different between Year 2 and Year 7, with slightly lower grades for Year 7 (t(66) = 3.20, p<0.05 Cohen’s d = 0.39; see Table S3 for means and SDs). There were no correlations between: 1) Year grade in Chinese and spatial ability for Year 2 and Year 7; and 2) the growth in spatial ability and changes in Year grade

| **Table S2 Pearson's Correlations across Year Grades and Spatial ability** | | | | | | | | | | | | | | | | | | | | | | | | | | | | | |
| --- | --- | --- | --- | --- | --- | --- | --- | --- | --- | --- | --- | --- | --- | --- | --- | --- | --- | --- | --- | --- | --- | --- | --- | --- | --- | --- | --- | --- | --- |
| **Variable** | |  | | YG_Rus_Year 2 | | YG_Rus_Year 7 | | YG_Math_Year 2 | | YG_Math_Year 7 | | YG_Eng_Year 2 | | YG_Math_Year 7 | | YG_Span_Year 2 | | YG_Span_Year 7 | | YG_Chin_Year 2 | | YG_Chin_Year 7 | | SA_Year 2 | | SA_Year 7 | | Delta_SA | |
| YG_Rus_Year 7 |  | n |  | 257 |  | — |  |  |  |  |  |  |  |  |  |  |  |  |  |  |  |  |  |  |  |  |  |  |  |
|  |  | r |  | .50 | *** | — |  |  |  |  |  |  |  |  |  |  |  |  |  |  |  |  |  |  |  |  |  |  |  |
| YG_Math_Year 2 |  | n |  | 281 |  | 257 |  | — |  |  |  |  |  |  |  |  |  |  |  |  |  |  |  |  |  |  |  |  |  |
|  |  | r |  | .72 | *** | .41 | *** | — |  |  |  |  |  |  |  |  |  |  |  |  |  |  |  |  |  |  |  |  |  |
| YG_Math_Year 7 |  | n |  | 253 |  | 255 |  | 253 |  | — |  |  |  |  |  |  |  |  |  |  |  |  |  |  |  |  |  |  |  |
|  |  | r |  | .43 | *** | .62 | *** | .41 | *** | — |  |  |  |  |  |  |  |  |  |  |  |  |  |  |  |  |  |  |  |
| YG_Eng_Year 2 |  | n |  | 205 |  | 185 |  | 205 |  | 181 |  | — |  |  |  |  |  |  |  |  |  |  |  |  |  |  |  |  |  |
|  |  | r |  | .60 | *** | .50 | *** | .59 | *** | .47 | *** | — |  |  |  |  |  |  |  |  |  |  |  |  |  |  |  |  |  |
| YG_Math_Year 7 |  | n |  | 238 |  | 237 |  | 238 |  | 234 |  | 174 |  | — |  |  |  |  |  |  |  |  |  |  |  |  |  |  |  |
|  |  | r |  | .52 | *** | .64 | *** | .44 | *** | .60 | *** | .57 | *** | — |  |  |  |  |  |  |  |  |  |  |  |  |  |  |  |
| YG_Span_Year 2 |  | n |  | 84 |  | 81 |  | 84 |  | 77 |  | 83 |  | 68 |  | — |  |  |  |  |  |  |  |  |  |  |  |  |  |
|  |  | r |  | .56 | *** | .45 | *** | .54 | *** | .31 | ** | .55 | *** | .53 | *** | — |  |  |  |  |  |  |  |  |  |  |  |  |  |
| YG_Span_Year 7 |  | n |  | 82 |  | 83 |  | 82 |  | 80 |  | 81 |  | 71 |  | 77 |  | — |  |  |  |  |  |  |  |  |  |  |  |
|  |  | r |  | .37 | *** | .58 | *** | .38 | *** | .54 | *** | .46 | *** | .60 | *** | .46 | *** | — |  |  |  |  |  |  |  |  |  |  |  |
| YG_Chin_Year 2 |  | n |  | 73 |  | 70 |  | 73 |  | 70 |  | 0 |  | 63 |  | 0 |  | 0 |  | — |  |  |  |  |  |  |  |  |  |
|  |  | r |  | .57 | *** | .43 | *** | .52 | *** | .44 | *** | NA |  | .53 | *** | NA |  | NA |  | — |  |  |  |  |  |  |  |  |  |
| YG_Chin_Year 7 |  | n |  | 72 |  | 73 |  | 72 |  | 73 |  | 5 |  | 68 |  | 0 |  | 0 |  | 67 |  | — |  |  |  |  |  |  |  |
|  |  | r |  | .41 | *** | .66 | *** | .39 | *** | .55 | *** | NA |  | .72 | *** | NA |  | NA |  | .46 | *** | — |  |  |  |  |  |  |  |
| SA_Year 2 |  | n |  | 188 |  | 176 |  | 188 |  | 172 |  | 126 |  | 164 |  | 61 |  | 58 |  | 61 |  | 55 |  | — |  |  |  |  |  |
|  |  | r |  | .09 |  | .04 |  | .18 | * | .04 |  | .17 |  | .01 |  | .36 | ** | .09 |  | .18 |  | -.04 |  | — |  |  |  |  |  |
| SA_Year 7 |  | n |  | 185 |  | 175 |  | 185 |  | 174 |  | 155 |  | 162 |  | 67 |  | 65 |  | 28 |  | 31 |  | 121 |  | — |  |  |  |
|  |  | r |  | .08 |  | .17 | * | .29 | *** | .17 | * | .12 |  | .11 |  | .30 | * | .16 |  | .11 |  | .11 |  | .15 |  | — |  |  |  |
| Delta_SA |  | n |  | 121 |  | 117 |  | 121 |  | 116 |  | 98 |  | 110 |  | 48 |  | 48 |  | 22 |  | 21 |  | 121 |  | 121 |  | — |  |
|  |  | r |  | -.02 |  | .07 |  | .14 |  | .10 |  | .07 |  | .09 |  | -.02 |  | .09 |  | -.12 |  | -.10 |  | -.44 | *** | .82 | *** | — |  |
| YG_Chin_Delta |  | n |  | 67 |  | 67 |  | 67 |  | 67 |  | 0 |  | 62 |  | 0 |  | 0 |  | 67 |  | 67 |  | 55 |  | 27 |  | 21 |  |
|  |  | r |  | .04 |  | .37 | ** | .03 |  | .28 | * | NA |  | .38 | ** | NA |  | NA |  | -.30 | * | .70 | *** | -.18 |  | .04 |  | -.04 |  |
|  | | | | | | | | | | | | | | | | | | | | | | | | | | | | | |
| Note: * p < .05, ** p < .01, *** p < .001; SA Growth was computed by subtracting SA Year 2 from SA Year 7; Chinese Year Grade Delta was computed by subtracting Chinese Year Grade for Year 2 from Chinese Year Grade for Year 7. | | | | | | | | | | | | | | | | | | | | | | | | | | | | | |

| **Table S3 Descriptive Statistics for year grades** | | | | | | | | | | | | | | | | | | | | | | | | | | | | | | | | | | | | | | | | | | | | | | | | | | | | | | | | | | | | | |
| --- | --- | --- | --- | --- | --- | --- | --- | --- | --- | --- | --- | --- | --- | --- | --- | --- | --- | --- | --- | --- | --- | --- | --- | --- | --- | --- | --- | --- | --- | --- | --- | --- | --- | --- | --- | --- | --- | --- | --- | --- | --- | --- | --- | --- | --- | --- | --- | --- | --- | --- | --- | --- | --- | --- | --- | --- | --- | --- | --- | --- | --- |
|  | | **YG_Rus_Year 2** | | | | | | **YG_Rus_Year 7** | | | | | | **YG_Math_Year 2** | | | | | | **YG_Math_Year 7** | | | | | | **YG_Eng_Year 2** | | | | | | **YG_Math_Year 7** | | | | | | **YG_Span_Year 2** | | | | | | **YG_Span_Year 7** | | | | | | **YG_Chin_Year 2** | | | | | | **YG_Chin_Year 7** | | | | | |
|  | | **1** | | **2** | | **3** | | **1** | | **2** | | **3** | | **1** | | **2** | | **3** | | **1** | | **2** | | **3** | | **1** | | **2** | | **3** | | **1** | | **2** | | **3** | | **1** | | **2** | | **3** | | **1** | | **2** | | **3** | | **1** | | **2** | | **3** | | **1** | | **2** | | **3** | |
| Valid |  | 114 |  | 87 |  | 80 |  | 96 |  | 86 |  | 77 |  | 96 |  | 82 |  | 77 |  | 114 |  | 87 |  | 80 |  | 112 |  | 86 |  | 7 |  | 98 |  | 71 |  | 70 |  | 2 |  | 82 |  | 0 |  | 0 |  | 83 |  | 0 |  | 0 |  | 0 |  | 73 |  | 0 |  | 0 |  | 73 |  |
| Mean |  | 3.84 |  | 4.29 |  | 4.14 |  | 3.59 |  | 4.08 |  | 3.78 |  | 3.51 |  | 3.63 |  | 3.70 |  | 3.85 |  | 4.46 |  | 4.29 |  | 4.20 |  | 4.40 |  | 4.43 |  | 3.92 |  | 4.04 |  | 3.87 |  | 3.50 |  | 4.54 |  | - |  | - |  | 4.36 |  | - |  | - |  | - |  | 4.34 |  | - |  | - |  | 4.01 |  |
| Std. Deviation |  | 0.67 |  | 0.59 |  | 0.52 |  | 0.67 |  | 0.67 |  | 0.58 |  | 0.61 |  | 0.71 |  | 0.63 |  | 0.67 |  | 0.52 |  | 0.53 |  | 0.71 |  | 0.64 |  | 0.79 |  | 0.77 |  | 0.76 |  | 0.72 |  | 0.71 |  | 0.53 |  | - |  | - |  | 0.71 |  | - |  | - |  | - |  | 0.56 |  | - |  | - |  | 0.72 |  |
| Minimum |  | 3.00 |  | 3.00 |  | 3.00 |  | 2.00 |  | 3.00 |  | 3.00 |  | 2.00 |  | 2.00 |  | 3.00 |  | 3.00 |  | 3.00 |  | 3.00 |  | 3.00 |  | 3.00 |  | 3.00 |  | 3.00 |  | 3.00 |  | 3.00 |  | 3.00 |  | 3.00 |  | - |  | - |  | 3.00 |  | - |  | - |  | - |  | 3.00 |  | - |  | - |  | 2.00 |  |
| Maximum |  | 5.00 |  | 5.00 |  | 5.00 |  | 5.00 |  | 5.00 |  | 5.00 |  | 5.00 |  | 5.00 |  | 5.00 |  | 5.00 |  | 5.00 |  | 5.00 |  | 5.00 |  | 5.00 |  | 5.00 |  | 5.00 |  | 5.00 |  | 5.00 |  | 4.00 |  | 5.00 |  | - |  | - |  | 5.00 |  | - |  | - |  | - |  | 5.00 |  | - |  | - |  | 5.00 |  |
| Note: Language variable is coded as follows: 1 – English only; 2 – English and Spanish; and 3 – English and Chinese | | | | | | | | | | | | | | | | | | | | | | | | | | | | | | | | | | | | | | | | | | | | | | | | | | | | | | | | | | | | | |
